# Lifestyle Factors Associated With Childhood Obesity in South Asian Expatriates of Dubai: A Cross-Sectional Study

**DOI:** 10.7759/cureus.74924

**Published:** 2024-12-01

**Authors:** Spriha Pandey, Meenu Agarwal

**Affiliations:** 1 Basic Sciences, GEMS Modern Academy, Dubai, ARE; 2 Pediatrics, Aster Jubilee Medical Center, Dubai, ARE

**Keywords:** cross-sectional studies, expatriates, lifestyle factors, obesity and overweight, school children

## Abstract

Background

Obesity, a chronic disease caused by excessive fat deposits, increases the risk of various health conditions. Childhood obesity is a growing global concern, affecting millions of children. There is a paucity of research on the determinants of childhood obesity in the expatriate population of Dubai. This study aimed to explore modifiable key lifestyle factors contributing to childhood obesity in Dubai's South Asian expatriates.

Methodology

Based on prior research, we decided to evaluate the role of three most commonly associated lifestyle factors: physical activity, fast food consumption, and daily screen time. The hypothesis was that higher screen time and fast food intake would positively correlate with body mass index (BMI), while physical activity would show a negative correlation. The study applied a non-probability convenience sampling method and included 208 South Asian children aged 5-17 who visited a Dubai clinic between January and March 2024 and who did not have any serious physical or mental health illness requiring long-term medication or any genetic disorder.

Results

Results showed that one-third of the children had a BMI of ≥85 percentile with no significant gender differences. Age was positively correlated with BMI percentile. Higher daily screen time is significantly associated with a higher prevalence of overweight and obesity as per the Centers for Disease Control and Prevention (CDC), International Obesity Task Force (IOTF), and World Health Organization (WHO) classifications. The frequent intake of fast food is associated with a higher prevalence of overweight and obesity only as per the IOTF classification. Physical activity, however, was not significantly different between the two groups.

Conclusion

We concluded that children who have a BMI of ≥85 percentile tend to have significantly higher daily screen time. The association between frequent fast food consumption and higher BMI was significant only under the IOTF criteria. Larger multicentric studies are needed to further validate these associations and to look for causal relationships. Nevertheless, this study underscores modifiable risk factors for childhood obesity, suggesting that small lifestyle changes could mitigate this epidemic.

## Introduction

Obesity is a chronic, multifaceted disease marked by excessive fat accumulation that can detrimentally affect health [1]. In the last few decades, obesity prevalence has been increasing not only in adults but also in children. In the long term, childhood obesity heightens the risk of developing hypertension, dyslipidemia, type 2 diabetes, coronary heart disease, stroke, gallbladder disease, osteoarthritis, sleep apnea, and breathing problems [1]. Obesity is also a significant contributor to mental health conditions among adolescents and teenagers such as anxiety, depression, body dysmorphic disorder, and eating disorders, particularly anorexia and bulimia [2]. A few studies have reported that childhood obesity is associated with a significantly increased risk for cardiovascular events in adult life, even if body weight had meanwhile normalized [3,4].

Childhood overweight and obesity are now being characterized as a medical epidemic, especially as its prevalence has been steadily increasing over the past few decades. In 2022, it was estimated that 37 million children under the age of five, along with over 390 million children and adolescents aged 5-19, were overweight. Globally, the obesity rates in children and adolescents aged 5-19 years have risen from 2% in 1990 to 8% in 2022 [1] with the World Health Assembly global nutrition targets advocating for "no rise in childhood overweight/obesity by 2025" [5]. To achieve this goal, it is vital to explore obesity's prevalence among various ethnicities and nationalities, regionally, nationally, and internationally, along with the key factors driving these trends.

Approximately 68% of the United Arab Emirates (UAE) population is made up of South Asian expatriates, with Indians forming the largest group [6]. In comparison to other ethnic groups, children of South Asian ancestry have a higher likelihood of developing obesity and associated metabolic risks [7]. Only one published study has explored the prevalence of childhood obesity among these expatriates, and it found that approximately one-third of school-aged Indian expatriates in the UAE were overweight, obese, or severely obese [8]. These rates were significantly higher than those found among Indian residents. In view of the findings of Shah et al. that male South Asian expatriates living in UAE for a longer duration are more likely to have diabetes, the higher prevalence of childhood obesity in Indian expatriates living in UAE in comparison to Indian residents would likely be due to lifestyle factors [9]. Since South Asians including Indians share a common socio-cultural and genetic predisposition for obesity, our study aims to investigate the impact of key lifestyle factors contributing to childhood obesity in South Asian expatriates living in Dubai [10]. Lifestyle factors can be significantly influenced by policy interventions, such as public awareness campaigns, which can play a key role in addressing the obesity epidemic. Based on some previous research, we identified three most prominent potentially modifiable lifestyle-related factors likely influencing childhood obesity: physical activity, frequency of fast food consumption, and daily screen time [11-13]. We hypothesized that daily screen time and fast food consumption frequency would have a strong positive correlation with body mass index (BMI), while the degree of physical activity would show a strong negative correlation with BMI in children.

## Materials and methods

This study was conducted at a Dubai-based medical clinic (Aster Jubilee Medical Center), and the study obtained approval from the clinic's Institutional Review Board and Ethics Committee; vide approval number AJMC/MOM/EC/23/026 dated December 18, 2023. We applied a non-probability convenience sampling method for this study. Convenience sampling was justifiable in our situation as it increased the cooperation of parents, children, and clinic staff for data collection. The sample included children of South Asian origin in the age range of five years to 17 years, who visited the pediatric department of the clinic between January and March 2024 and who did not have any serious medical illness requiring long-term medication. The differently abled children, children suffering from mental health conditions and children with genetic disorders, and children with chronic illnesses such as malignancy, tuberculosis, hormonal disorders such as precocious puberty, diabetes, and thyroid disorders were excluded from the study. The children who were younger than five years of age were also not included in the study. For all children who agreed to be part of this study, the assents of children and written informed consents of parents were obtained.

Based on the abovementioned inclusion and exclusion criteria, a total of 208 children were included in this study. The demographic and anthropometric data of the children was charted by licensed nurses of the clinic. The age, gender, height, and weight of the children were charted. Age was calculated as the number of completed years. Standing height was gauged using sturdy stadiometers, with the children maintaining an erect posture and barefooted. Weight was assessed using precise balance scales, while children were attired in light dresses and without footwear. All measurements were taken and recorded by Dubai Health Authority (DHA)-registered nurses, endorsed by the Ministry of Health, under the supervision of a licensed pediatrician.

Additionally, parents were asked to fill up a questionnaire (Appendices). The questionnaire was pre-piloted for face validity by distributing it to 10 parents of children of the targeted age, and their comments and recommendations were taken into consideration in the final version of the survey, but their responses were included in the results. The questionnaire consisted of three questions. The first question was as follows: how frequently the child plays a sport that involves vigorous physical activity such as running, jumping, or swimming? Examples of such sports were mentioned on the questionnaire and included tennis, swimming, running, basketball, cricket, gymnastics, and wrestling. Physical activity scores were graded from 1 to 4 as follows: always (score: 4), 6-7 days/week; often (score: 3), 4-5 days/week; sometimes (score: 2), 2-3 days/week; and rarely, (score: 1), 0-1 days/week. The second question was as follows: how frequently the child takes fast food or high-caloric sweetened beverages? Examples of such food items were given to the parents and included ice cream, candy, chocolate, desserts, canned fruit juices, sugary canned drinks, and packaged food items. Fast food intake scores were given on a scale of 1-4 using the same frequency scale as for physical activity scores. The third question enquired about the daily screen time of children, which parents responded based on their recall. Screen time was scored on a similar scale of 1-4 as follows: a score of 1 for screen time of <1 hour a day, a score of 2 for 1-2 hours of screen time per day, a score of 3 for 2-4 hours of screen time per day, ana d score of 4 for >4 hours of screen time each day.

The BMI of all children was calculated using the standard formula (weight in kg/height in meters squared). Gender-specific US growth charts were employed to classify children as overweight (BMI of ≥85th percentile), obese (BMI of ≥95th percentile), or extremely obese (BMI of ≥99th percentile) according to the World Health Organization (WHO), International Obesity Task Force (IOTF), and Centers for Disease Control and Prevention (CDC) standards. All BMI calculations were performed using a website (http://cmhsweb.uaeu.ac.ae/childbmicalculator) developed by the Ministry of Health, UAE [14].

Statistical analysis

The presentation of the categorical variables was done in the form of numbers and percentages. On the other hand, the quantitative data with normal distribution was presented as the mean ± SD and the data with non-normal distribution as median with 25th and 75th percentiles (interquartile range). The data normality was checked by using the Shapiro-Wilk test. In the cases in which the data was not normal, we used nonparametric tests. The following statistical tests were applied to the results: (1) The comparison/association of the variables that were quantitative and not normally distributed in nature was analyzed using the Mann-Whitney test, and the variables that were quantitative and normally distributed in nature were analyzed using the independent t test; (2) the comparison/association of the variables that were qualitative in nature was analyzed using the chi-square test; and (3) the Spearman rank correlation coefficient was used for the correlation of age of children with CDC BMI percentile.

The data entry was done in the Microsoft Excel (Microsoft Corp., Redmond, WA) spreadsheet, and the final analysis was done with the use of the Statistical Package for Social Sciences (SPSS) software version 25.0 (IBM Corp., Armonk, NY).

For statistical significance, a p value of less than 0.05 was considered statistically significant.

## Results

A total of 208 expatriate children of South Asian origin participated in this study. The mean age of the children was 9.02 years, with a range of 5-17 years. Of all the participants, 124 (59.62%) were girls, and 84 (40.38%) were boys. This demographic distribution of our study sample has been summarized in Table 1.

**Table 1:** Demographic profile of the study population

Demographic characteristics	Frequency	Percentage
Gender		
Female	124	59.62%
Male	84	40.38%
Age (years)		
Mean ± SD	9.02 ± 3	
Median (25th-75th percentile)	8 (7-11)	
Range	5-17	

Prevalence of overweight, obesity, and extreme obesity

Based on the CDC, IOTF, and WHO guidelines, the children were divided into two groups: first, those who had a BMI of ≥85th percentile for age and gender; second, those who had a BMI of <85 percentile for age and gender. A BMI of ≥85th percentile for age and gender includes overweight, obese, and extremely obese children, and a BMI of <85 percentile includes normal-weight and underweight children. As per the CDC guidelines, a total of 64 children (30.77%) were overweight, obese, or extremely obese. Children with a BMI of ≥85th percentile were 73 (35.10%) and 61 (29.33%) by the WHO and IOTF criteria, respectively. IOTF underestimated and WHO overestimated overweight, obesity, and extreme obesity, while CDC values were between those of IOTF and WHO (Table 2). These results are in keeping with previously published series [14,15].

**Table 2:** Prevalence of overweight, obesity, and extreme obesity as per the CDC, IOTF, and WHO criteria CDC, Centers for Disease Control and Prevention; IOTF, International Obesity Task Force; WHO, World Health Organization; BMI, body mass index

CDC criteria		IOTF criteria		WHO criteria	
Underweight + normal weight (<85 percentile BMI)	Overweight + obese + extremely obese (≥85 percentile BMI)	Underweight + normal weight (<85 percentile BMI)	Overweight + obese + extremely obese (≥85 percentile BMI)	Underweight + normal weight (<85 percentile BMI)	Overweight + obese + extremely obese (≥85 percentile BMI)
144 (69.23%)	64 (30.77%)	147 (70.67%)	61 (29.33%)	135 (64.90%)	73 (35.10%)

Gender distribution of BMI of ≥85 percentile

**Figure 1:**
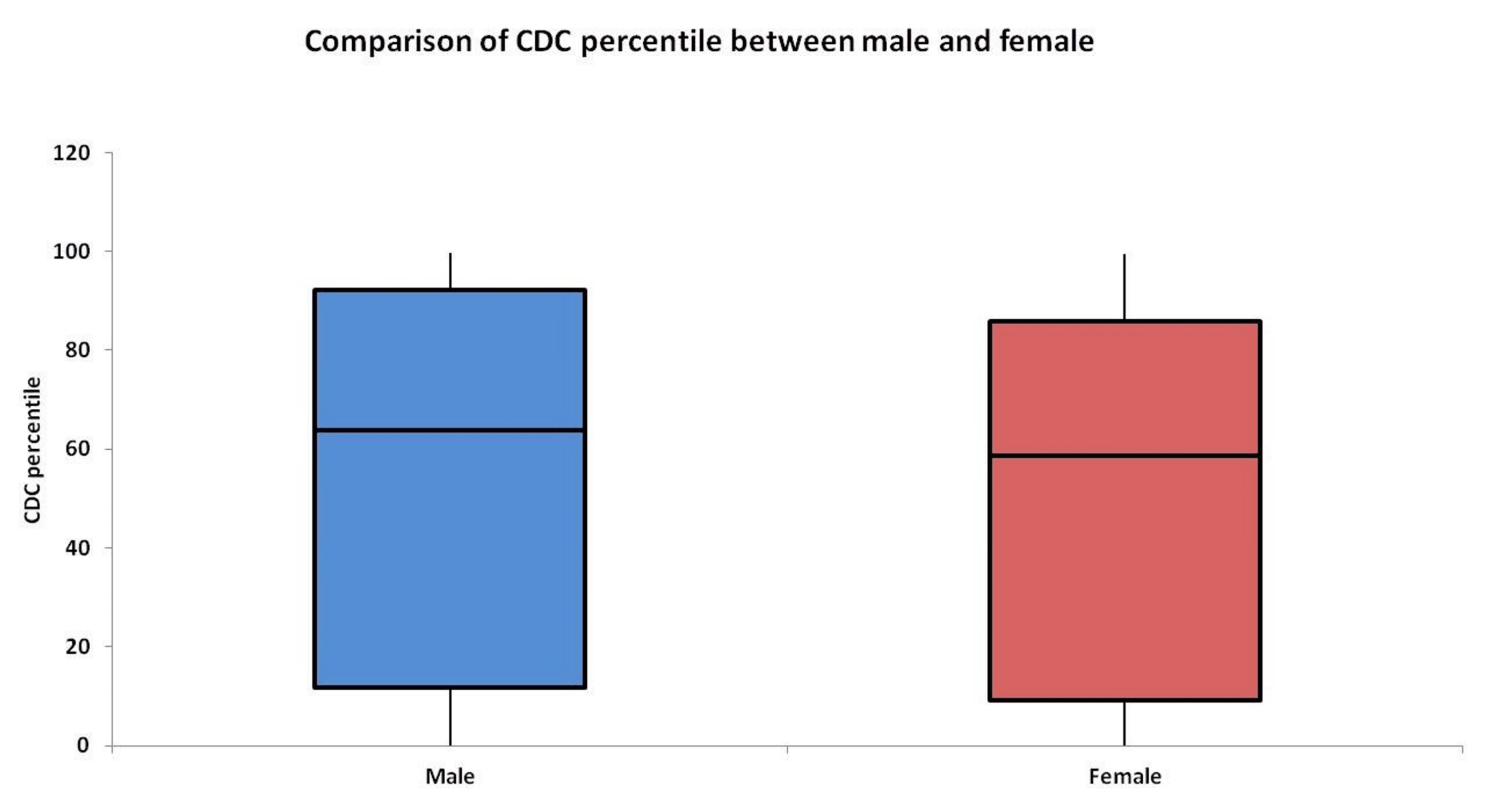
Comparison of CDC BMI percentile between boys and girls CDC, Centers for Disease Control and Prevention; BMI, body mass index

As per the CDC criteria, 36.90% of boys (31/84) and 26.61% of girls (33/124) were either overweight, obese, or extremely obese, and these rates were comparable in the boys and girls with no statistically significant difference (p = 0.115). Boys and girls had median BMI percentile of 63.85 and 58.65 by the CDC criteria, respectively, with no significant gender difference (p = 0.252) (Table 3 and Figure 1).

**Table 3:** Distribution of CDC BMI percentile in boys and girls †Mann-Whitney test CDC, Centers for Disease Control and Prevention; BMI, body mass index

CDC BMI percentile	Boys	Girls	Total	P value
Median (25th-75th percentile)	63.85 (11.7-92.2)	58.65 (9.25-85.825)	61.55 (9.45-89.175)	0.252^†^

Similar trends were also seen using the WHO and IOTF guidelines with BMI distribution being comparable in the two genders. Of the boys and girls, 33.33% and 26.61%, respectively, had a BMI of ≥85 percentile according to the IOTF criteria (p = 0.296). Similarly, the prevalence of BMI of ≥85 percentile in boys and girls was 40.48% and 31.45%, respectively, by WHO cutoffs (p = 0.181) (Table 4).

**Table 4:** Comparison of overweight and obesity rates between boys and girls *Chi-square test CDC, Centers for Disease Control and Prevention; IOTF, International Obesity Task Force; WHO, World Health Organization

	Boys (n = 84)	Girls (n = 124)	Total boys and girls (n = 208)	P value
CDC classification				
<85 percentile	53 (63.10%)	91 (73.39%)	144 (69.23%)	0.115^*^
≥85 percentile	31 (36.90%)	33 (26.61%)	64 (30.77%)	
IOTF classification				
<85 percentile	56 (66.67%)	91 (73.39%)	147 (70.67%)	0.296^*^
≥85 percentile	28 (33.33%)	33 (26.61%)	61 (29.33%)	
WHO classification				
<85 percentile	50 (59.52%)	85 (68.55%)	135 (64.90%)	0.181^*^
≥85 percentile	34 (40.48%)	39 (31.45%)	73 (35.10%)	

Correlation of BMI percentile by CDC criteria with age

**Figure 2:**
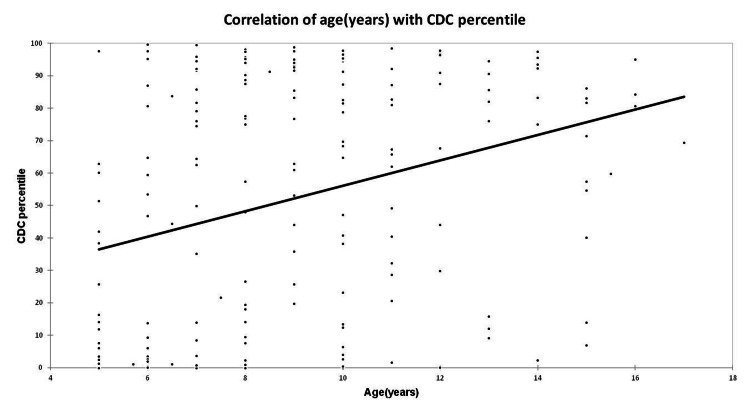
Correlation of age (in years) with CDC BMI percentile CDC, Centers for Disease Control and Prevention; BMI, body mass index

We found that there was a significant positive correlation (Spearman rank correlation coefficient, 0.314; p < 0.0001) of BMI percentile by CDC criteria with the age of children. As the age increases, there are significant chances of increase in the BMI percentile (Figure 2).

Association of physical activity, fast food, and screen time with the CDC classification

The screen time score was significantly higher in the first group (2.73 ± 0.96) of children with a BMI of >85 percentile in comparison to the second group (2.39 ± 0.93) comprising normal-weight and underweight children (p = 0.015). The physical activity and fast food intake scores were comparable in these two groups with no statistically significant difference (Table 5).

**Table 5:** Association of physical activity, fast food, and screen time with BMI of children ‡Independent t test CDC, Centers for Disease Control and Prevention; IOTF, International Obesity Task Force; WHO, World Health Organization; BMI, body mass index

CDC classification	<85th percentile (n = 144)	≥85th percentile (n = 64)	Total	P value
Physical activity	2.3 ± 0.79	2.41 ± 0.9	2.33 ± 0.82	0.385^‡^
Fast food	2.12 ± 0.77	2.31 ± 0.87	2.18 ± 0.81	0.123^‡^
Screen time	2.39 ± 0.93	2.73 ± 0.96	2.5 ± 0.95	0.015^‡^
IOTF classification	<85th percentile (n = 147)	≥85th percentile (n = 61)	Total	P value
Physical activity	2.29 ± 0.78	2.44 ± 0.92	2.33 ± 0.82	0.246^‡^
Fast food	2.11 ± 0.78	2.36 ± 0.86	2.18 ± 0.81	0.04^‡^
Screen time	2.41 ± 0.93	2.7 ± 0.97	2.5 ± 0.95	0.041^‡^
WHO classification	<85th percentile (n = 135)	≥85th percentile (n = 73)	Total	P value
Physical activity	2.3 ± 0.78	2.38 ± 0.89	2.33 ± 0.82	0.505^‡^
Fast food	2.11 ± 0.77	2.32 ± 0.86	2.18 ± 0.81	0.094^‡^
Screen time	2.38 ± 0.95	2.71 ± 0.92	2.5 ± 0.95	0.015^‡^

Association of physical activity, fast food, and screen time with the IOTF classification

The mean ± SD of fast food intake score in children with a BMI of <85th percentile was 2.11 ± 0.78, while it was 2.36 ± 0.86 in children with a BMI of ≥85th percentile (p = 0.04). Similarly, the mean ± SD of screen time score was 2.41 ± 0.93 in children with a BMI of <85th percentile, while it was 2.7 ± 0.97 in children with a BMI of ≥85th percentile (p = 0.041). Hence, the children who were overweight, obese, or extremely obese had significantly higher screen time and fast food intake scores. However, the physical activity scores were comparable in the two groups with no statistically significant difference (Table 5).

Association of physical activity, fast food, and screen time with the WHO classification

The mean ± SD of screen time was 2.38 ± 0.95 in children with a BMI of <85th percentile, while it was 2.71 ± 0.92 in children with a BMI of ≥85th percentile (p = 0.015), which concludes that screen time was significantly higher in overweight, obese, and extremely obese children compared to the normal weight and underweight children. However, the physical activity and fast food intake scores were comparable in these two with no statistically significant difference (Table 5).

## Discussion

South Asian Americans are predisposed to abdominal obesity and have disproportionately high rates of cardiovascular disease compared to other racial and ethnic groups in the United States [10]. Similarly, children of South Asian ancestry have a higher likelihood of developing obesity and associated metabolic risks [7]. Along with the genetic factors, socio-cultural factors such as high consumption of fried snacks, sweets, and high-fat dairy and the lack of leisure-time physical activity also play a role in the higher risk of obesity in South Asians [10]. There are many published studies evaluating childhood obesity in South Asian immigrants living in Western countries [7,16,17]. In contrast, there is a paucity of studies about South Asian expatriates living in Middle Eastern countries. Approximately 8.5 million South Asian expatriates live in UAE, comprising approximately 68% of the total UAE population with the Indian expatriates being the largest community accounting for approximately 37.96% of the total UAE population [6].

Moreover, there is a large population of South Asian expatriates living in other Middle Eastern Gulf countries. The Indian expatriates form the largest South Asian community in the Middle East with approximately nine million Indians currently living in the countries of the Gulf Cooperation Council. Despite such a large South Asian expatriate population, there is only one published study that found that the childhood obesity rate was much higher in the Indian expatriates of Dubai in comparison to the Indians living in India [8]. The lifestyle of these South Asian expatriates is quite different from their counterparts living in their home countries. Our study is the first study exploring the lifestyle determinants of childhood obesity in South Asian expatriates living in Dubai. We particularly focused on those lifestyle factors that can be easily modified by specific interventions. In our study cohort, approximately one-third of children were overweight, obese, or extremely obese. The interesting findings that emerge from this study include the following: (1) The prevalence of overweight and obesity is comparable in boys and girls with no statistically significant difference; (2) as the age of children increases, the prevalence of overweight and obesity increases; (3) higher daily screen time is significantly associated with the higher prevalence of overweight and obesity as per the CDC, IOTF, and WHO classifications; (4) frequent intake of fast food is associated with the higher prevalence of overweight and obesity as per the IOTF classification, but no statistically significant association is seen as per the CDC and WHO classifications; and (5) the physical activity of children is not significantly associated with the prevalence of overweight and obesity.

The various factors that predispose an individual child to gain excess body weight are complex and include biological factors such as genetics and perinatal influences, as well as lifestyle factors such as a sedentary lifestyle including physical inactivity, long screen time such as watching television or spending time on social media and dietary factors such as high and frequent intake of fast food and high-caloric sweetened beverages. There are various research linking genetic and perinatal factors with childhood obesity; however, the evaluation of these biological determinants of childhood obesity was out of the scope of this study [18,19]. In the current study, we primarily focused only on the lifestyle factors that influence child BMI because these factors can be potentially modified to tackle the increasing prevalence of childhood obesity. Since today's children spend a lot of time on digital screens for entertainment, networking, and even for education, it was not surprising that in our study, daily screen time was significantly more in overweight and obese children by the CDC, WHO, and IOTF classifications. Our result was similar to US-based studies, which found that almost half of obese children engaged in more than two hours a day of screen time, compared to almost one-third of normal-weight children [20,21]. A strong association of childhood obesity with screen time was also found in previously published studies done in Middle Eastern countries [22,23]. Based on these findings, we can conclude that daily screen time is an important potentially modifiable component of the sedentary behavior of children, which needs to be addressed to control childhood obesity.

Although the hypothesis of the inverse association of physical activity in children with childhood obesity is intuitively logical, the results of previously published series have not always been corroborating. Bener et al. found that less physical activity was a significant independent predictor of adolescent obesity in Qatar [22]. Similarly, Al-Hazzaa et al. showed that overweight/obesity was significantly and inversely associated with vigorous physical activity levels [24]. Mahumud et al. found that the lack of physical activity and an excessively sedentary lifestyle were highly associated with the risk of developing obesity in adolescents [25]. McGavock et al. found that children with low cardiorespiratory fitness had a significantly higher risk of being overweight [26]. In contrast to these results, Al-Kloub et al. found that the level of physical activity was not associated with adolescent obesity [27]. Similarly, Jildeh et al. did not find any association of physical activity with obesity in Palestinian adolescents [28]. In our study cohort, the physical activity score in overweight and obese children was comparable to the normal-weight and underweight children with no statistically significant difference between the two groups. This may be because of the small sample size. Another reason may be that since parents were asked to subjectively tell the weekly frequency of active involvement of children in sports requiring vigorous physical effort, they might have under- or overestimated the physical activity. Moreover, parents may not be fully aware of their children's physical activity during school hours.

Along with a sedentary lifestyle, dietary factors such as high and frequent intake of fast/junk food are considered an important risk factor for childhood obesity. A meta-analysis by Malik et al. concluded that there was strong evidence that the consumption of sugar-sweetened beverages was associated with excessive weight gain [29]. In a large study, Mahumud et al. reported that those consuming more than two times carbonated soft drinks and at least one fast food daily were at a substantially higher risk of being overweight or obese [25]. In our study cohort, the children who were overweight, obese, or extremely obese by the IOTF classification had significantly higher fast food intake scores. However, when we applied the WHO or CDC classification, children with a BMI of ≥85th percentile had higher fast food intake scores, but these scores were comparable with normal-weight and underweight children, and the differences between the two groups were not statistically significant. The likely explanation for such disparity is the small sample size.

This study has some limitations. Primarily, this study is based on a small sample selected from a single medical clinic using convenience sampling. The study determined the rate of obesity based on BMI, which tends to underestimate body fat percentage in South Asian children; however, we applied the WHO, IOTF, and CDC BMI criteria, which are the most validated and widely used for measuring childhood obesity. This study primarily explored lifestyle-related determinants of childhood obesity as these can be potentially modifiable and can guide effective policy interventions. The genetic, perinatal, and socio-economic factors influencing the children's weight were not studied. In addition, other factors such as negative parenting characteristics, poor sleep pattern, and emotional stress were not examined in the current study. Physical activity, screen time, and fast food consumption were scored only subjectively by the parents, and this might have introduced a bias. However, a study by Otten et al. found that self-reported measures of television viewing were significantly correlated with an objective electronic television monitor and had a high level of agreement; thus, self-reporting is not necessarily a major limitation [30]. One important limitation of this study is that being cross-sectional in design, this study did not delve into the changing rate of obesity over time. Though the results of this study are significant in revealing the association of lifestyle factors with childhood obesity, a causal relationship cannot be inferred based on this study. Larger multicentric and longitudinal studies should be done to better evaluate the independent predictors of childhood obesity in the expatriate population.

## Conclusions

This study is the first study to explore the potentially modifiable lifestyle factors associated with childhood obesity in South Asian expatriates living in Dubai. The obesity rates are comparable in boys and girls. As the age increases, the children tend to have higher BMI. In our study cohort, the mean daily screen time has emerged as the most consistent factor associated with childhood obesity. The result of this study bears imminent relevance as it indicates that small alterations in a child's lifestyle such as restricting screen time to a set number of hours, observing a screen-free hour before sleeping, and adhering to defined screen-free zones and screen-free periods can go a long way to control childhood obesity. The parents, teachers, and children need to be sensitized to the urgent need to regulate daily screen time.

Interestingly, in this study, the association between fast food consumption and higher BMI was significant only under the IOTF criteria. Similarly, our study did not find any significant association of physical activity with childhood obesity. Since this study is based on a small sample from a single clinic, larger multicentric longitudinal studies should be done to further evaluate and validate these potential associations and causal relationships.

## Appendices

Table 6 shows the questionnaire filled up by parents. 

**Table 6:** Questionnaire

Question	Score: 1	Score: 2	Score: 3	Score: 4
Q1: How frequently child plays a sport that involves vigorous physical activity such as running, jumping, or swimming? Examples of such sports include tennis, swimming, running, basketball, cricket, gymnastics, and wrestling.	Rare (0-1 day)	Sometimes (2-3 days)	Often (4-5 days)	Always (6-7 days/week)
Q2: How frequently the child takes fast food or high-caloric sweetened beverages? Examples of such food items include ice cream, candy, chocolate, desserts, canned fruit juices, sugary canned drinks, and packaged food items.	Rare (0-1 day)	Sometimes (2-3 days)	Often (4-5 days)	Always (6-7 days/week)
Q3: How many hours the child uses electronic gadgets per day? Examples of electronic gadgets include TV, computer, tablet, and mobile phone.	<1 hour	1-2 hours	2-4 hours	>4 hours
